# Discussion on Soldering

**Published:** 1897-09

**Authors:** 


ARTICLE VI.

### DISCUSSION ON SOLDERING.

Dr. E. D. Swain: In 1861, when I left my patients temporarily, after a practice of five or six years in different parts of the country, every man knew how to use the blowpipe. Every man knew how to solder. Not only that, but to make his own plate and to make his own solder. I presume Dr. Cushing remembers very well the difficulties he had in learning to use the blowpipe. I do. But when it came to the idea of keeping up a continuous blast, it was very sudden, and from then until now it is one of the things we cannot forget. I was out of the profession for nearly five years, and when I returned to practice such a thing as soldering was hardly known in a dental office in the northern part of this country, nor even the southern part. There was no occasion to use the blowpipe. Consequently the condition of things which Dr. Peck saw in a dental college had only then come about as a result. The men who were educated between 1861 and the time when crowns and bridge-work first began to be made were not taught to solder, but are now to some extent teachers and demonstrators. There was hardly anything but vulcanite except in a few offices, and the dentists who occupied those offices were considered old fogies. Metals were not used, but the introduction of gold crowns and of bridge-work came about, and the working back again into metal for partial plates to a considerable extent has brought about a change, and a change for the better without question.

After listening to Dr. Peck's paper and seeing the demonstrations which he has made, I feel that he has left very little for me to discuss. Of course, the members of the society might give us some points as to their particular mode of operating, but as my own method is so near like that described by Dr. Peck, I have very little to say upon the subject except to remark that in order to do a good piece of soldering, after making everything clean, and even sometimes taking the trouble to scrape the surface on which you are to apply the flux, you should heat the investment and the plate and teeth first almost, if not quite, down to the flowing of the solder, and then directing the blaze upon the soldering; it drops down to its place· smoothly, making the use of the chisel almost unecessary Another thing which is accomplished by that is that the solder always flows toward the hottest point, and if the investment is heated beneath and behind the teeth themselves, the solder flows into the interstices and makes good solid work. The solder flows under and between the backing in the plate and makes it strong between. If you attempt to make it solder otherwise, and the flame is put to the solder too early, it produces a sort of sweating process, in jewelers' parlance; the piece of solder is fused sufficiently to adhere or cohere.

I do not know that I can give you any new points in regard to soldering. Cleanliness from begining to end is one of the things which should always be looked after. It is poor policy, if not dangerous, to attempt to solder a piece of work that has not first been boiled in pickle or in alum water, in order that the oxide which forms from the alloy in previous heatings shall be removed. Either that should be done, or the plate and backing should be scraped to give a clean surface.

Dr. A. O. Hunt: I agree with Dr. Swain that Dr. Peck has been so painstaking and careful in regard to the smallest details in this demonstration of soldering without investment that there is very little to be discussed. However, there was one thought came to me at the time when he mentioned the unequal expansion of platinum and porcelian. It is this: that if a large piece of solder is laid over all, so that if, at any time during the process of rotating the flam in, under and about the investment, it cannot come in direct contract with the platinum pins, causing the procelain to check. A plate of heavier solder prevents the too rapid transition of heat to the metal underneath, and thus equalizes more nearly the expansion of the metal and porcelain.

I was very much pleased with the demonstration of soldering with porcelain not invested. It is well known that porcelain in the process of fusing has to withstand a very much higher degree of heat than that required for the solder to flow. But very few would always be sufficiently painstaking and careful about their methods of applying the heat to the porcelain if the crown or piece was not invested. For this reason the investment is of some value; but it is not absolutely essential, as Dr. Peck has demonstrated.

I feel like saying a few words in the line of Dr. Swain's remarks. From 1861, or perhaps a year or two earlier than that—from 1859 to 1860—up to 1880, there was hardly any metal work done that required soldering by those entering the profession during that period. If gold was used as the base of the denture, the soldering was not of a particular character; it consisted merely in the soldering of loops on a plate to retain rubber or celluloid. This was before bridge-work was introduced into the profession to any extent. For twenty years or more the delicate manipulation of metals and porcelain combined was a lost art in dental practice. The experience gained before that time in the art of using the mouth blowpipe skillfully will never be forgotten. I prefer the bellows. There are features in the use of the latter that I regard as as important. With the blowpipe in the mouth, the head must be at a fixed distance from the flame nearly all the time. One hand is occupied in directing the blowpipe, while in using the bellows both hands and the head are free, so that one is enabled to see any part of the work to better advantage. A continuous flame can be managed by the bellows with so little effort that I am one of those who believe it is a decided improvement over the mouth blowpipe. One other point is with reference to the kinds of flame to be used. A fine reducing flame of the blowpipe is rarely ever called for in joining the points of a crown or bridge. The brush or larger flame, which will cover the greater part of the piece to be soldered, is more desirable, as one is less likely to break the porcelain, the heat being more evenly distributed over a larger area.

Dr. W. H. Taggart: I wish to commend Dr. Peck's paper, and if I am wrong in the criticism I am going to make, I wish the doctor would draw my attention to it. It is this: In the crown that he soldered for us, the parts were simply united with the solder, but the places where the solder would want to be flushed in to contour it, or in additional places where we desire to bring out the full contour of the tooth, I believe he did it with the flame.

Dr. Peck: Yes, I did. I shall have occasion to do it again in my office to-morrow.

Dr. Taggart: While Dr. Peck does that, it seems to me it is much harder to do it that way than to draw the solder to one point with the blow-pipe flame. As. Dr. Swain has said, the heat will go to the hottest part, and there is a tendency for all the solder, when it has become melted, to gravitate to one point, leaving parts that are already soldered properly. You have to rotate it to get it to stay at one point. It is much easier to do this with the blow-pipe than with anything else.

Again, Dr. Peck spoke of putting on all pieces of solder at one time. I would ask him whether he thinks that is desirable.

Dr. Peck: Yes, I think it is in such a case as I described.

Dr. Taggart: It seems to me the fewer pieces of the solder you put on at one time the better you fuse it, and get it to flow into all places without forming pits or pockets. It would be an advantage to put on pieces as they melt, than to put on all pieces at one time. These are the only criticisms I would make.

Dr. F. N. Brown: Crown and bridge-work are my food and drink; I scarcely study anything else. I do not proceed exactly as the essayist. The first thing I do when I procure any teeth from the dental depot, is to remove every particle of wax and allow none to come in contact with my work while in process of construction. I use "Parr's wax flux" in waxing up and holding parts together until the piece is invested. It is then placed over a Bunsen heater and the "Parr's wax flux" burns out, leaving the piece fluxed. I then sprinkle it with a little dry powdered borax, and place enough solder on to cover the pins of the teeth. I then proceed to heat the investment and solder up, adding one piece of solder at a time. I never remove my piece of work to a soldering block, or anything else, but let it remain on the Bunsen heater while soldering up.

Dr. Peck says, All breakages come from backings being so positioned as to put strain on the porcelain in cooling." I think most breakages come from allowing the porcelain facings to touch, and that from expansion the facings are cracked. In soldering, I never use the mouth blowpipe; I prefer a blowpipe on a stand, so that I can keep a foot-bellows or compressed air apparatus going, then having the flame constantly directed on the work.

In this way there is no cessation of heat at any time. The use of "Parr's wax flux" in bridge-work cannot be overvalued.

(Here Dr. Brown demonstrated on the blackboard.)

Dr. B. J. Cigrand: I desire to say a few words in reference to Dr. Peck's paper. It is a paper that I think will do both the older and younger members a great deal of good. He has emphasized the unimportance of investment; we have learned that the investment is for the purpose of only holding the parts in apposition and position while soldering; whereas in single small pieces, where the parts can be held in apposition by wire or otherwise, investment is not necessary. The lesson taught us this evening I learned some years ago under somewhat exciting circumstances. When I first started to practice dentistry, one of my first cases called for the application of a bridge to restore the teeth. While I had the bridge and large investment on the fire, my attention was directed to the fact that I had some errand to do down town. I went down town to one of the dental depots, unintentionally left my investment and bridge on the fire at the office, and did not return for about two hours. While I was down town it occurred to me that I had left my bridge and investment on the fire, and you may be sure it did not take me very long to catch the first car. When I reached my office I believed that the bridge was spoiled, and as I am somewhat of an optimist, I tried to make the best of it. I made up my mind that the bridge was spoiled. I saw that it was red-hot and thoroughly radiated, and after using the blow-pipe a little, the solder flowed away like so much cream.

I was determined, in the event of a failure, to have the personal pleasure of having brought it about, executed my intent, little thinking that an underlying principle in soldering would be revealed. I threw the case in powdered plaster of Paris to cool, and, upon disinvesting the case, I found I had made one of the best solders in my life; the secret of which is: use the blow-pipe very little and get the case good and hard. I hardly ever use a blow-pipe except in large bridges. I use the Haskell-Bunsen burner, and I never employ a blowpipe in the production of what we call barrel or telescope crowns. I make the entire operation with a Bunsen burner.

Another point Dr. Peck has shown us is how to prevent the solder from flowing in the directions where he does not want it to flow, and to attain this he uses ordinary stove blacking. I employ instead of this agent a paste of whiting and rouge, and find it fully answering the purpose.

When I make a barrel or telescope crown, after I have made the band and readjusted the cusps, and invert it and proceed to solder it to the band, the band may, while in the flame, become unsoldered. If the stove blacking or whiting, whichever you prefer, is used on the joint of the band, you can solder the cusp full of gold, and there will be no unsoldering or opening up of the band. I have recently resorted to the method of filing all my solders as they come from the dental depots into almost a dust; in order to eliminate all the steel, etc., from the filings, I pass through it a large magnet; this way your solders will melt a good deal quicker, since you create a greater surface for the flame to play on. With reference to holding the crown in the flame and the likelihood of the solder jumping out of position on account of the puckering up of the borax, if we take the crown and use gum arabic, making it into a mucilage over the borax, we can force or hold the solder wherever we like, and it will stick there.

I believe Dr. Peck has emphasized the true underlying principles of soldering better than I have ever heard or read of. Briefly, I would again say, get your case well heated and seldom use the blow-pipe.

Dr. J. H. Woolley: I was glad Dr. Cigrand brought out a point concerning borax which is not touched upon very frequently. Borax in the process of melting has a tendency to move the solder. If the borax is melted previous to its use and ground finely, it will not interfere with the solder, so that that process makes the placing of the solder just where it naturally would belong, or where it was needed.

Dr. Peck (closing the discussion). One point referred to by Dr. Hunt, that of placing large pieces of solder over the pins to prevent them from becoming unduly heated, may be of service in some cases, but if the investment is thoroughly heated from the under side first, as indicated in the paper, such precautions as the one cited above are positively unnecessary.

As regards the use of the bellows, I do not object to it. Indeed, to those who are in the habit of using it I would say, Never give it up. I do not care to use it, because I have become so thoroughly accustomed to the mouth blow-pipe that I use it in all cases without the slightest difficulty or fatigue, and, too, I believe I have better control of the flame than one can possibly have when using the bellows.

This crown can be as easily contoured in the Bunsen flame as if it were invested. The solder can be melted on one side to any extent desired without disturbing that on the other side in the slightest. It may be easier for some to invest and reinvest for the purpose of contouring, but not for me.

It may not be out of place to state that the crown I made in clinic before the State society last spring was heated and cooled five different times for the purpose of perfecting the soldering and contouring, which also demonstrated the fact that there is no possible danger of breakage with this manner of soldering.

The "correction" cited by Dr. Taggart, that in soldering large cases very small pieces of solder should be melted into the crevices, etc., after which more is added from time to time until the case is completed, I consider one of the greatest mistakes one can make in doing this work. What is the use of occupying the extra time necessary to permit the case to partially cool, to place more solder, and to reheat, and to repeat the foregoing several times until the soldering is completed, when all can be done in most cases with one heating. All that is necessary is care in the preparation of the case for soldering; the crevices must be thoroughly cleansed and fluxed and the case thoroughly heated from the under side. If this preparatory work is not thoroughly done, one must not expect desirable results.

However, you will remember I stated in the paper that we have our individual methods of producing results, and we are oftentimes very prone to consider our method the best. It matters little to me what method one uses, provided he uses it well and gains the desired result within a reasonable time.

I do not think the teeth or facings in contact one with the other are any more liable to break than when they are spaced—providing the case is evenly and thoroughly heated. Many times I have soldered large cases in which the teeth were in close contact one with the other; in fact, in some instances even jointed together, and I haven't gotten into trouble. I can't help believing that the predominating cause of the breakage of teeth and facings is the untimely heating of the pins.—*Dental Review.*

---

